# *Pneumocystis jirovecii* pneumonia in non-HIV patients: need for a more extended prophylaxis

**DOI:** 10.3389/fmed.2024.1414092

**Published:** 2024-06-26

**Authors:** Léo Sauvat, Laure Denis, Céline Nourrisson, Philippe Poirier, Marc Ruivard, Guillaume Le Guenno

**Affiliations:** ^1^Infection Control Department, CHU Clermont-Ferrand, Clermont-Ferrand, France; ^2^Department of Internal Medicine, CH Alpes - Lemans, Contamine sur Arve, France; ^3^Laboratory of Mycology and Parasitology, Centre Hospitalier Universitaire (CHU) Clermont-Ferrand, 3IHP, Clermont-Ferrand, France; ^4^Department of Internal Medicine, Centre Hospitalier Universitaire (CHU) Clermont-Ferrand, Clermont-Ferrand, France

**Keywords:** pneumocystis, immunosuppression, prophylaxis, guidelines, TMP-SMX

## Abstract

**Background:**

*Pneumocystis jirovecii* pneumonia (PCP) has a significant mortality rate for non-HIV immunocompromised patients. Prevention is primarily based on combined trimethoprim and sulfamethoxazole (TMP-SMX) but guidelines on pneumocystosis prophylaxis are scattered and not consensual.

**Objectives:**

This study aims to describe PCP in non-HIV patients and to review case by case the prior indication of prophylaxis according to specific guidelines.

We included patients with confirmed diagnosis of PCP admitted to one university hospital from 2007 to 2020. Prior indication for pneumocystis prophylaxis was assessed according to the specific guidelines for the underlying pathology or treatment.

**Results:**

Of 150 patients with a medical diagnosis of PCP, 78 were included. Four groups of underlying pathologies were identified: hematological pathologies (42%), autoimmune diseases (27%), organ transplantation (17%), and other pathologies at risk of PCP (14%). A small subgroup of 14 patients (18%) had received a prior prescription of pneumocystis prophylaxis but none at the time of the episode. Transfer to intensive care was necessary for 33 (42%) patients, and the mortality rate at 3 months was 20%. According to international disease society guidelines, 52 patients (59%) should have been on prophylaxis at the time of the pneumocystis episode. Lowest compliance with guidelines was observed in the hematological disease group for 24 patients (72%) without prescription of indicated prophylaxis.

**Conclusion:**

Infectious disease specialists should draw up specific prophylactic guidelines against pneumocystis to promote a better prevention of the disease and include additional criteria in their recommendations according to individual characteristics to prevent fatal cases.

## Introduction

*Pneumocystis jirovecii* pneumonia (PCP) was first described in association with human immunodeficiency virus (HIV) caused by the opportunistic fungus *Pneumocystis jirovecii* (1). Originally, patients with PCP were attributed with acquired immunodeficiency syndrome (AIDS) status. The disease occurs when CD4+ T lymphocytes cells are below 200 cells/mm^3^ (2). The standard prophylaxis is mainly based on combined trimethoprim and sulfamethoxazole (TMP-SMX) or atovaquone, dapsone, pentamidine until sufficient immune recovery (3). At a prophylactic dose, TMP-SMX exceptionally leads to the prescription being suspended because of adverse effects, mainly caused by leukopenia (3.1% of cases) (4).

One major current challenge is the increasing occurrence of PCP in non-HIV immunocompromised patients as a result of specific diseases, immunosuppressive treatments or chemotherapies (5). In Germany, the incidence of PCP significantly increased from 2014 to 2019 as well as PCP-related mortality (+19%) on population level (6). The spectrum of high-risk underlying diseases includes hematological malignancies, solid tumors, inflammatory diseases and solid organ transplants (SOT). The most frequent risk factors recognized are hematologic malignancies, chronic lung diseases and non-hematological cancers. Guidelines focusing on PCP prevention are available in hematology (7–9) and in SOT (10–12) but not in internal medicine except for autoimmune inflammatory rheumatic disease (13). Although prescription of PCP prophylaxis is recommended, it is inconsistently promoted by clinicians. This is of concern given the high mortality of the disease, which can reach up to 46%, much higher than that observed for HIV patients (14).

To improve PCP prevention, we performed a retrospective study of non-HIV patients infected by PCP to analyze underlying pathologies and treatments at risk of PCP and to evaluate the theoretical recommendation for prophylaxis on a case-by-case basis according to international guidelines.

## Materials and methods

This was an observational study of patients admitted for PCP at the university hospital of Clermont-Ferrand, France, from 01/01/2007 to 01/15/2020. The patients were identified from the databases of the hospital and the mycology laboratory using the International Classification of Diseases (ICD-10) associated with the diagnosis of PCP.

Inclusion criteria were age over 18 years, clinical features suggestive of PCP (dyspnea, fever, cough) with a compatible radiological pattern on CT scan, and presence of microbiological evidence of PCP. Exclusion criteria were HIV seropositivity and patients under 18 years of age. During data collection, each patient file was analyzed to confirm the ICD-10 diagnosis of PCP registered by the medical practitioner.

For microbiological detection, sampling sites included bronchoalveolar lavage fluid (BAL) or induced sputum. Microbiological diagnosis of PCP was confirmed by identification of *P. jirovecii* by Gomori-Grocott (GG) and/or May-Grünwald Giemsa (MGG) staining method or by in-house quantitative polymerase chain reaction (qPCR) (15). Patients were included regardless of the cycle threshold (Ct) value of the qPCR. Because of changes in PCR techniques over the years, and the variability of BAL performance, the PCR replication cycle was not analyzed. The hypothesis of possible colonization was ruled out by a strict review of the file with clinical and radiological data indicative of PCP.

For each patient, disease history, chemotherapy and/or immunosuppressive treatments, and the clinical, radiological, and biological characteristics of PCP were collected. CT scan reports were used as diagnostic confirmation of PCP. Previous prescriptions of prophylaxis (TMP-SMX, atovaquone, dapsone and pentamidine) and its interruption were investigated in correspondence concerning each patient. The indication for prophylaxis was evaluated for each case according to the specific guidelines of the underlying pathology (Supplementary Tables S1, S2) available at the date of the PCP. Adverse effects of TMP/SMX were registered by the practitioners managing the patient and recorded as such if considered to be the reason for discontinuing prophylaxis. Mortality data were obtained from local medical records and death notices. Patients were informed at the time of hospitalization of the possibility of using their data in an anonymous register and the study was declared to the National Commission for Information Technology and Civil Liberties (registration number I2017).

### Statistical analysis

Continuous variables are presented as medians or means and interquartile ranges, and categorical variables as counts and percentages.

## Results

### Patient characteristics

**Figure 1:**
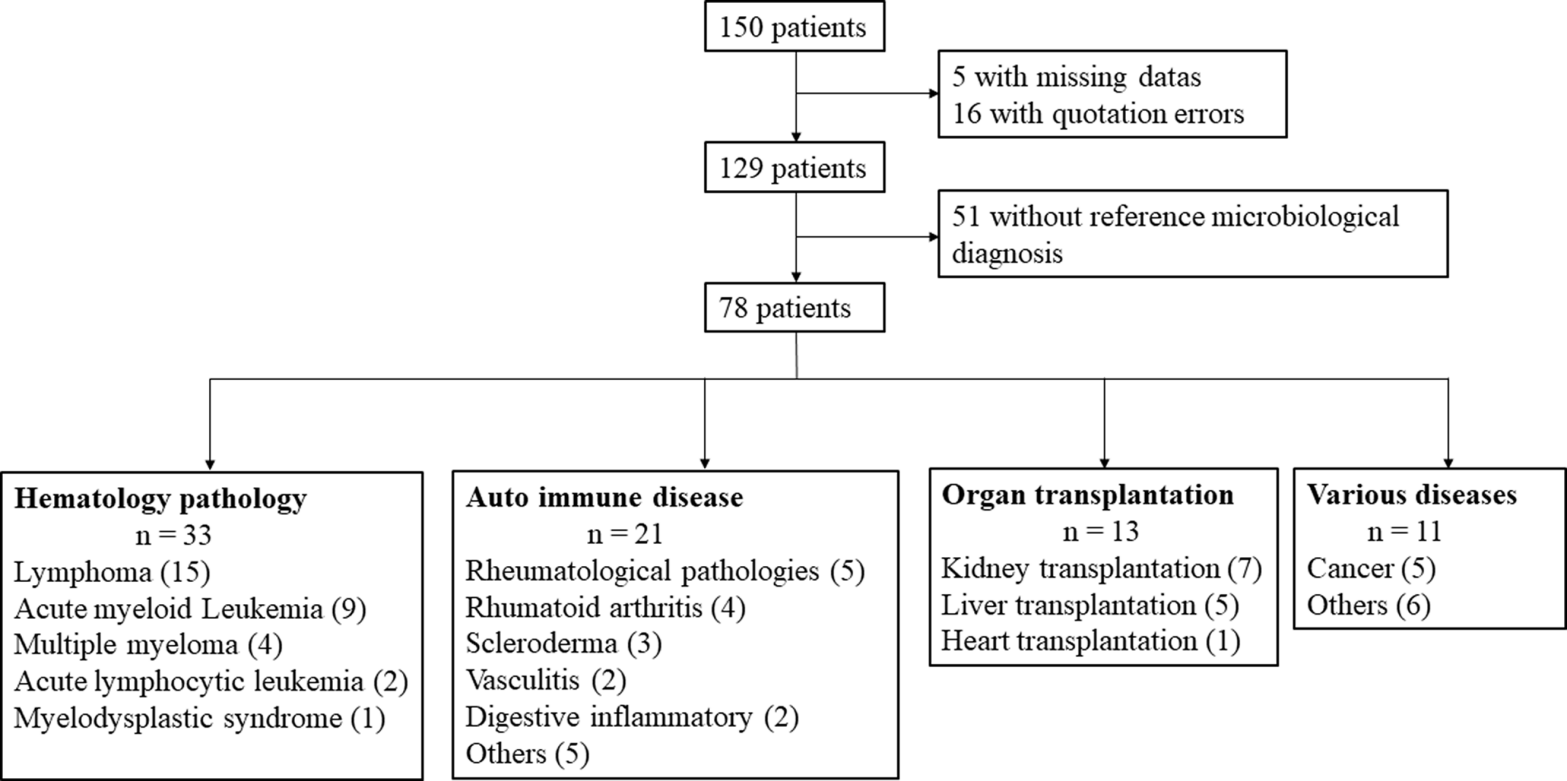
Flow chart of patients with PCP diagnosis and classification into four groups: hematological malignancy, autoimmune disease, solid organ transplantation, and others pathologies.

After review of the 150 records containing a PCP diagnosis, 21 patients were excluded because of missing data or scoring errors (Figure 1). Of the remaining 129 cases, 51 were not included owing to the lack of mycological evidence (absence of positive *P. jirovecii* qPCR). Hence, the study involved a total of 78 patients who were classified into four groups (Table 1): hematological malignancy, autoimmune disease (AID), solid organ transplantation (SOT), and the other remaining diseases (Figure 1).

**Table 1:** Characteristics of patients and underlying pathology at risk of *Pneumocystis jirovecii* pneumonia (PCP) in the four groups: hematological malignancy, autoimmune disease (AID), solid organ transplantation and other pathologies.

	Total	Hematological malignancy	AID	Solid organ transplantation	Others
n (%)	78	33 (42)	21 (35)	13 (17)	11 (14)
Age (mean, years)	67.0	64.8	68.2	60.5	66.1
Male gender (*n* (%))	48 (62)	19 (58)	13 (62)	6 (41)	10 (91)
Smoking^a^ (*n* (%))	18 (23)	6 (18)	5 (24)	4 (31)	3 (27)
Pulmonary disease (*n* (%))^b^	16 (21)	1 (3)	3 (14)	4 (31)	8 (73)
Corticosteroids (*n* (%))	29 (37)	4 (12)	12 (57)	8 (62)	5 (45)
Corticosteroids (mean in mg)	13	4	21	5	27
Chemotherapy or treatment at risk of PCP (*n* (%))	66 (85)	30 (91)	11 (52)	13 (100)	11 (92)
Treatment duration^c^ (median in months [IQR])	9.3 [2, 92]	4.8 [1, 40]	56.9 [3, 120]	86.6 [43, 200]	3.3 [2, 8]
Time from first treatment at risk of PCP^c^ (median in months [IQR])	16.6 [4, 106]	11 [3, 39]	56.9 [3, 119]	56.5 [39, 200]	5.1 [2, 8]

a Previous or active smoking. (notion present in the medical record).

b Chronic Obstructive Pulmonary Disease, asthma, fibrosis and/or chronic respiratory insufficiency.

c Corticosteroids, chemotherapy, immunosuppressive therapy.

In the hematology group (*n* = 33; 42%), 6 (18%) patients had the PCP episode after autologous transplantation and 3 (10%) after allogeneic transplantation (of whom 1 had an autologous transplant followed by an allogeneic transplant). In the AID group (*n* = 21, 27%), several risk factor diseases were listed among “Others”: one case each of autoimmune hepatitis, autoimmune hemolytic anemia, dermatomyositis, myasthenia, and microscopic polyangiitis. In the SOT group (*n* = 13; 17%), 7 (54%) patients had a kidney graft as risk factor, 5 (38%) a liver graft and 1 (8%) a heart graft. In the remaining diseases group “Other diseases” (*n* = 11; 14%) with the exception of 5 cancer patients (45%), we included one case each of cordarone-induced interstitial lung disease, idiopathic pulmonary fibrosis, Cushing’s disease, meningioma, hyperthyroidism and leishmaniasis with hemophagocytic syndrome treated with Etoposide.

### Indication for prior PCP prophylaxis according to guidelines

Forty-six patients (59%) had a previous indication for a prophylactic treatment according to the guidelines. In the solid organ transplant group, 2 patients should have been on prophylaxis because engraftment was less than 12 months old. In the “other pathologies” group, 5 (42%) patients were treated with corticosteroids at more than 20 mg daily, and 1 patient because of Cushing’s disease. In the AID group, 9 patients had a recommendation for prophylaxis because the dosage of corticosteroid therapy was higher than 20 mg, and 5 patients were treated with methotrexate. In the hematological disease group, 24 patients (72%) had a recommendation for prophylaxis, 11 because of the pathology (9 cases of acute myeloid leukemia and 2 of acute lymphoid leukemia), 5 because of autograft, 3 treated with rituximab, cyclophosphamide, doxorubicin hydrochloride vincristine sulfate and prednisone regimen (RCHOP), 2 with high doses of methotrexate, 1 with rituximab combined with fludarabine and cyclophosphamide (R-FC), and 2 treated with high doses of dexamethasone for myeloma.

Only 14 patients (21%) had previously received prophylaxis and had discontinued it before the episode of PCP. Of these, 7 discontinued without reason, 2 because of non-indication for hematological pathologies, 1 for renal failure, 1 because more than a year had elapsed since transplantation, 1 for pancytopenia, 1 following agranulocytosis, and 1 for mild elevated transaminase levels.

### Characteristics of the pneumocystosis episode

Dyspnea was the major symptom (83%). According to the New York Heart Association (NYHA) functional classification, 43 patients (55%) belonged to stage IV, and 11 (14%) to stage II-III. Fever was present in 51 patients (65%) on admission to hospital and cough in 38 (49%) (Table 2). Thirty-nine patients (50%) had crackles on pulmonary auscultation. After reading of the pulmonary CT scan, the involvement was considered typical if a symmetrical peripheral ground-glass involvement was observed. Of the 75 patients undergoing thoracic CT–scan, 67 (86%) had ground-glass opacities, which were bilateral in 62 (82%) and associated with alveolar condensation in 18 (23%).

**Table 2:** Characteristics of the clinical, biological and radiological presentation at the diagnosis of *Pneumocystis jirovecii* pneumonia (PCP) in the different groups of pathologies.

	Total	Hematology	AID	Transplantation	Others
*n* (%)	78	33 (42)	21 (35)	13 (17)	11 (14)
Acute form* (*n* (%))	36 (46)	14 (42)	10 (48)	7 (54)	5 (45)
Fever (*n* (%))	52 (67)	27 (84)	16 (76)	7 (54)	2 (18)
Pulmonary crackles (*n* (%))	41 (53)	13 (41)	14 (67)	6 (46)	8 (72)
Stage IV NYH (*n* (%))	43 (55)	14 (42)	12 (57)	6 (46)	11 (100)
Nosocomial PC (*n* (%))	12 (15)	11 (33)	1 (5)	0 (0)	0
Typical pulmonary CT-scan (*n* (%))	62 (79)	30 (94)	16 (76)	9 (69)	7 (64)
Bronchial fibroscop (number [%])	76 (97)	33 (100)	20 (95)	13 (100)	11 (100)
MGG and/or GG stainings + (*n* (%))	10 (13)	1 (3)	3 (14)	4 (31)	2 (18)
PCR PCP LBA + (*n* (%))	77 (99)	33 (100)	20 (95)	13 (100)	11 (100)
Co-infection					
Virus + (number [%])	8 (10)	6 (18)	1 (5)	1 (77)	0
PCR CMV + (*n* (%))	4 (5)	0	2 (1)	1 (8)	1 (10)
Other pathogens (*n* (%))	28 (36)	11 (33)	9 (43)	4 (31)	4 (36)
Biological findings					
Leukocytes (G/L) (median [IQR])	6.9 [4; 11]	4.4 [2; 7]	10.5 [7; 12]	5.8 [4; 12]	12 [8; 15]
Lymphocytes (G/L) (median [IQR])	0.6 [0,3; 1,0]	0.4 [0,2; 07]	0.9 [0,7; 1,8]	0.4 [0,3; 1,0]	0.9 [0,6; 1,2]
Gammaglobulins (g/l) (median [IQR])	7.8 [4,5; 12]	9 [6; 14]	7.6 [5; 10]	6 [6; 12]	9.8 [8; 11]
LDH (U/L) (median [IQR]) (Standard: 87–241)	297 [250; 374]	273 [246; 362]	383 [294; 492]	291 [230; 351]	271 [259; 339]
CRP (mg/L) (median [IQR])	93 [56; 141]	111 [80; 148]	92 [79; 134]	83 [60; 234]	39 [19; 116]

AID, Autoimmune disease; CT-scan, computed tomography scan; MGG/GG, May-Grünwald Giemsa/Gomori-Grocott staining; NYHA, New York Heart Association. *Acute form (clinical form evolving within 48 h).

Regarding the mycological diagnosis, light microscopy (MGG and/or GG stainings) was positive in 10 patients (13%) and PCR pneumocystis-positive in all of them (for one patient, diagnosis was performed on induced sputum with specific PCR). Colonizers and pathogens were searched on BAL and other specific microbiological samples (sputum and nasopharyngeal swab notably). Respiratory viruses (Influenza virus A/B, VRS, Rhinovirus-Enterovirus, Adenovirus, Coronavirus, MERS-CoV, Metapneumovirus, Parainfluenza virus) (FilmArray, BioFire), HSV-1, HSV-2, VZV (RealStar alpha Herpesvirus PCR Kit 1.0, Altona Diagnostics), HHV6 (RealStar Adenovirus PCR Kit 1.0, Altona Diagnostics) and CMV (CMV R-gene, Argene)) were searched on BAL in 73 patients and were positive in 8 (3 cases of HSV-1, 3 of VRS, and 2 of rhinovirus). Bacteria, detected either by culture: 1 *Staphylococcus epidermidis*, 4 *Pseudomonas aeruginosa,* 1 *Morganella morganii*, 1 *Escherichia coli,* 1 *Acinetobacter baumanii*, 1 *Enterobacter cloacae complex,;* or by urinary antigen test: 1 *Streptococcus pneumoniae*), viruses (3 HSV-1, 3 VRS, 2 Rhinovirus, 1 HHV6, 1 Influenza virus; all detected by PCR), and fungi (detected by culture: 5 *Aspergillus* sp., 6 *Candida albicans*, 1 *Candida tropicalis*, 1 *Candida glabrata,* 1 *Cladosporium* sp., 1 *Cryptococcus neoformans* and 1 *Penicillium* sp.) were also found.

The pre-event (at least 3 months before) lymphocyte count median was 0.8 G/L (IQR, 0.4; 1.3) in 53 patients. The median CD4 lymphocyte T cell count at the time of the episode was 286/mm^3^ (IQR, 149; 723) in 14 patients and the median PaO2 was 60 mmHg (IQR, 52; 69) in the 33 patients who had an arterial gasometry.

Management of the pneumocystosis episode and clinical course are described in Table 3. Sixty-six (85%) patients were treated in first line with TMP-SMX, of which 40 patients were treated intravenously. Ten patients (13%) were treated with Wellvone in first line and 8 patients in second line. One patient was treated with Pentacarinat and Wellvone and 1 patient with only Pentacarinat and TMP-SMX in second line. Side effects caused by TMP-SMX included renal failure in 17 patients (21%), liver dysfunction in 2 (3%), cytopenia in 2 (2%) and other non-specific complications in 6 patients (8%). Forty-four patients (56%) were treated for co-infection without systematic microbiological evidence, 40 patients (51%) were co-treated with at least 1 antibiotic, 11 patients (14%) were treated with antifungal agents and 3 (4%) with an antiviral agent. Concerning short-term mortality (during PCP episode) or long-term mortality (at 3 months), two (3%) deaths were directly related to the worsening of the respiratory infection, three (4%) to the worsening of the main underlying pathology and nine (12%) were due to several factors.

**Table 3:** Management and outcome of the *Pneumocystis jirovecii* pneumonia (PCP) in the different groups of pathologies.

	Total	Hematology	AID	Transplantation	Others
*n* (%)	78	33 (42)	21 (35)	13 (17)	11 (14)
First line TMP-SMX therapy	65 (83)	27 (64)	20 (95)	8 (61)	10 (91)
Combined anti-infective treatments	46 (59)	21 (64)	11 (52)	7 (54)	7 (63)
Good tolerance	51 (66)	23 (70)	13 (62)	6 (46)	9 (82)
Corticosteroid therapy	29 (37)	12 (36)	8 (38)	5 (38)	4 (36)
Intensive care unit	33 (42)	14 (42)	11 (52)	4 (31)	4 (36)
Favorable outcome*	55 (78)	25 (78)	13 (61)	13 (100)	4 (36)
Secondary prophylaxis	40 (51)	21 (66)	8 (38)	7 (54)	4 (36)
Death at 3 months	16 (20)	5 (15)	5 (24)	2 (15)	4 (36)

AID, autoimmune disease group; TMP-SMX, trimethoprim-sulfamethoxazole, *Favorable outcome = Good evolution of PCP during treatment without additional infectious, respiratory complication or death.

## Discussion

In our present study, forty-six (59%) patients should have been protected by pneumocystosis prophylaxis at the time of the PCP episode. Quite alarmingly, 20 patients (34%) were not prescribed secondary prophylaxis after the acute episode. It clearly emerges that clinicians lacked knowledge of the guidelines about PCP prevention and thus had difficulties in strictly following them. The proportion of patients with underlying hematological pathology in PCP cases is high (16). The clinical features remain non-specific with dyspnea being the main clinical symptom as previously reported (14) and normal pulmonary auscultation in half of the patients. Hence, a thoracic CT scan was needed to look for bilateral ground-glass opacities in immunocompromised patients as evidence of typical lung involvement [16]. The 3-month mortality rate was a concern at 20% (16 deaths). Pulmonary coinfection was reported in more than a third of the patients and was previously described as having a higher mortality risk when associated with CMV (17).

Follow-up guidelines have always been a challenge for prophylaxis. In a quality assessment, only 38% of 204 patients treated with immunosuppressive drugs had adequate prophylaxis prescribed for PCP according to local guidelines (18). Regarding immunocompromised patients with PCP, up to 50% of the patients should have a prior prophylaxis prescription according to international guidelines (19). In a specific hematopoietic cell transplantation cohort (20), 10% of the patients did not receive an initial prescription of PCP prophylaxis despite it being recommended according to standard protocol. In addition, only 45% of the patients had prophylaxis prescribed until the end of the at-risk period. The guidelines provided to the practitioners and used in this study (7–12) are disparate and could differ for the same underlying risk factor depending on the scientific society. Given the issue of their accessibility and consistency it has been suggested that, to improve adherence, guidelines need to be simplified by optimizing their design and making the content clear and easier to read (21). To incorporate new, relevant data, living systematic reviews are needed to provide up-to-date guidelines to target users (22).

Prescribing TMP-SMX as prophylaxis of PCP for immunocompromised, polypathological patients is a challenge. Further reasons for discontinuing prophylaxis with TMP-SMX are known: side effects leading to tolerance problems (digestive disorders in particular) and the fear of major side effects such as Steven-Johnson syndrome, agranulocytosis, aplastic anemia, and fulminant hepatic necrosis (23). In addition, polypharmacy can be a reason to not prescribe TMP-SMX to avoid drug–drug interaction such as inhibition of cytochrome P450 system and renal drug transporter inhibition, especially if TMP-SMX prophylaxis is prescribed in a specific medical context (chronic renal failure, hematological pathology). In internal medicine and inflammatory diseases, PCP prevention is based on historical studies without strong and clear guidelines. Treatments considered as incurring high risks of PCP are cyclophosphamide (24), high-dose steroids (25), and the association of immunosuppressors (26, 27). Unfortunately, many practitioners continue to refuse to prescribe the combination of TMP and SMX at a prophylactic dosage with low-dose methotrexate due to the fear of potentially cumulative hematotoxic side effects (28) despite studies showing that the combination carries no risk (29–31). The treatment at risk for which recommendations are least followed is corticosteroid therapy of more than 20 mg/day for 4 weeks, which is a risk factor highlighted in the literature (8, 9, 25) especially when it is combined with immunodepression or immunosuppressive therapy. The most important biological marker in guidelines to be considered as a risk factor of PCP is lymphopenia when the rate is lower than <1 G/L (32–34) and associated with a decrease in CD4^+^ T lymphocytes (35, 36). Risk is confirmed by a median level of lymphocytes of 0.6 G/L (0.3; 1.0). Thus, prophylaxis of PCP should be considered when corticosteroid therapy is prolonged and/or re-intensified, especially when combined with immunosuppressive therapy if the patient is elderly and has lymphopenia (37).

The period of prophylaxis of PCP is too short according to guidelines. In our population sample, there were 7 patients who had contracted PCP after a median period of more than 7 years after transplantation. PCP is known to occur late following transplantation at a median of 3 years whereas guidelines suggest an indication for prophylaxis up to 12 months (38). Hence, prescription rules for transplantation should not be restrictive and should be reassessed for each patient (39). In the case of hematology autograft patients, official recommendations provide no timeline for prophylaxis of PCP but the three episodes of PCP in our study occurring several years after the specific condition suggest it could be implemented later (11). One of the key findings of the study was the occurrence of PCP without indication for prophylaxis according to the guidelines. The group most concerned was solid organ transplantation: almost all patients (*n* = 12, 92%) had standard duration of prophylaxis which was discontinued. A major challenge for future guidelines will be to reassess potentially risky treatments, notably immunosuppressive therapy and the duration of prophylaxis, particularly for transplant patients. In general, once prophylaxis is prescribed, patient adherence to treatment is observed to be excellent at 18 months with a follow-up of over 80% for kidney transplant patients (40). From a more global perspective, risk assessment ranking can be used to take into consideration all these criteria depending on the underlying pathology (41, 42) and more specifically for rheumatoid arthritis (43). Going beyond the strict application of standard guidelines of international societies, the prophylaxis of PCP should be extended according to the criteria mentioned above and for a longer period. In addition, prescribers need to consider the evolution of at-risk pathologies. COVID-19 infection has been singled out as a major new risk factor for the same underlying pathologies or treatment (44). However, this disease can also occur in immunocompetent patients who have achieved a transitory state of immunosuppression (corticotherapy, lymphodepletion) during treatment with COVID-19 (45).

In our study, the two essential points of concern were the diagnosis of PCP and a review of the rules of the prescription of PCP prophylaxis. The strict inclusion criteria we used to confirm PCP based on microbiological evidence led to a restricted study population but avoided over-diagnosis for complex cases. All the files were checked to confirm the diagnosis of PCP in the data system to differentiate *P. jirovecii* colonization from infection because the main limitation of *P. jirovecii* PCR is that a positive result may be due to colonization. Concerning guidelines, changes in patient profiles could have occurred owing to the extended period of inclusion but this was offset by a follow-up of known updated guidelines according to the year of PCP diagnosis. Our study confirms the need for closer involvement of infectiologists in implementing a better follow-up of guidelines and raising awareness of complex cases. Specific meetings should be convened and national guidelines issued in conjunction with a regular update for the indication and duration of prescription for prophylaxis. In the era of interconnected systems, a simple, targeted method for identifying patients requiring PCP prophylaxis is emerging. Automatic monitoring of patients with a high-risk pathology or treatment, combined with analysis of biological criteria of interest such as lymphocytes associated with CD4^+^ T lymphocytes could alert the specialist directly. In addition, analysis of connected prescriptions would add an extra value to this personalized monitoring.

## Conclusion

PCP is a severe disease with a high mortality rate for non-HIV and immunocompromised patients. Compliance with guidelines must be improved with a systematic follow-up of prophylaxis guidelines for hematological diseases and organ transplantation. In autoimmune diseases, the difficulty of delivering comprehensive guidelines including all the complexities of specific pathologies is evidenced by the low rate of prior prescription. The issue that every clinician should consider for each patient with cellular immunodepression would seem to be the prescription of TMP-SMX prophylaxis. To go further, could these episodes of PCP have been avoided if the clinician had considered the indication for prophylaxis by looking beyond a conventional application of the guidelines? In our study, the prescription could have been discussed case-by-case for the 32 patients according to their clinical and biological characteristics and the treatments prescribed. The underlying pathology, ongoing therapies, and the persistence of lymphopenia should be assessed before ongoing prophylaxis is discontinued. A better follow-up and clearer guidelines with an extended prescription period are strongly recommended to prevent fatal cases of PCP. Infectious disease specialists should take part in the promotion of guidelines and be available for help in confirming prophylaxis in complex cases.

## Data availability statement

The raw data supporting the conclusions of this article will be made available by the authors, without undue reservation.

## Ethics statement

The studies involving humans were approved by Direction de la Recherche Clinique et Innovations CHU Clermont-ferrand. The studies were conducted in accordance with the local legislation and institutional requirements. The ethics committee/institutional review board waived the requirement of written informed consent for participation from the participants or the participants' legal guardians/next of kin because Patients were informed at the time of hospitalization of the possibility of using their data in an anonymous register and the study was declared to the National Commission for Information Technology and Civil Liberties (registration number 157 I2017).

## Author contributions

LS: Writing – original draft, Writing – review & editing, Conceptualization, Formal analysis, Investigation, Methodology. LD: Writing – review & editing, Conceptualization, Formal analysis, Investigation, Methodology. CN: Writing – review & editing, Conceptualization, Formal analysis, Investigation, Methodology. PP: Writing – review & editing, Conceptualization, Methodology. MR: Writing – review & editing, Conceptualization, Methodology. GG: Writing – original draft, Writing – review & editing, Conceptualization, Methodology, Supervision.
