# Supplementary material for: Pneumocystis jirovecii pneumonia in non-HIV patients: need for a more extended prophylaxis
Source: Front Med (Lausanne). 2024 Jun 26;11:1414092. doi: 10.3389/fmed.2024.1414092 (PMC11233525; doi:10.3389/fmed.2024.1414092)
Supplement: Supplementary file 1 [file Table_1.docx]

| Guidelines / Treatment - Pathology | Ann Hematol  (2013)(6) | Internal Medicine Journal (2014) (7) | European conference on infections in leukaemia (2016) (5) | American Thoracic Society (2010) |
| --- | --- | --- | --- | --- |
| Corticotherapy | **A**  Long term | **C**  16 mg or  dexa > 4mg during 4 weeks | **A**  (20 mg a day for 4 weeks) | 20 mg a day for at least 4 weeks |
| Acute lymphocytic leukemia | **A** | **A** | **A**  (induction to maintenance) | *Especially if*  *- T-cell deficiency*  *- Immunosuppressive treatment with a cytotoxic or anti-TNF treatment*  *- CD4 lymphocytes < 200 / mm3* |
| CD4 low count | **A** (< 200 ug/L) | **C** |  |  |
| BEACOPPesc | **C** |  |  |  |
| RCHOP | **C** | **C** |  |  |
| Prolonged neutropenia | **C** |  |  |  |
| Acute myelogenous leukemia | **C** |  |  |  |
| High dose cytarabine | **C** |  |  |  |
| Brain tumors (temozolomide, radiotherapy) |  | **B** |  |  |
| Allograft |  | **C** | **A** ≥ 6 months as long as immunocompromised |  |
| AML / Lymphoma (children) |  | **C** |  |  |
| Autograft |  | **C** |  |  |
| Methotrexate |  | **C** |  |  |
| Alemtuzumab |  | **C** (1 year) | **A** ≥ 6 months |  |
| Solid tumor (myelosuppressive chemotherapy for children) |  | **C** |  |  |
| FCR |  | **D** (1 year) |  |  |
| ABVD |  | **D** |  |  |
| Gemcitabine |  | **D** |  |  |

Supplementary Table 1: Level of evidence of practice guidelines for PCP prophylaxis. A: There is good research evidence to support the recommendation. B: There is fair research-based evidence to support the recommendation. C: The recommendation is based on expert opinion and panel consensus. BEACOPPesc = Bleomycin, Etoposide, Doxorubicin, Cyclophosphamide, Vincristine, Procarbazine and Prednisone, F- CR= Rituximab - Fludarabine – Cyclophosphamide, ABVD= Doxorubicin,Bleomycine, Vinblastine, Dacarbazine. AML= acute myelogenous leukemia
