# Supplementary material for: Pneumocystis jirovecii pneumonia in non-HIV patients: need for a more extended prophylaxis
Source: Front Med (Lausanne). 2024 Jun 26;11:1414092. doi: 10.3389/fmed.2024.1414092 (PMC11233525; doi:10.3389/fmed.2024.1414092)
Supplement: Supplementary file 2 [file Table_2.docx]

| Guidelines | Prophylaxis time |
| --- | --- |
| European Transplant Guidelines (2002) | 4 months |
| American Society of Transplantation (2009) | 6 to 12 months |
| Kidney Disease Improving Global Outcome (2010) / BMC Nephrology (2017) | 3 to 6 months |
| Transplant Infectious Disease (2011) | 6 to 12 months |
|  | Increased immunosuppression when rejection occurs |
|  | Chronic CMV infection |
|  | Prolonged corticosteroid therapy |
|  | Prolonged neutropenia |
|  | Autoimmune disease flare-ups |
|  | Prolonged contact with a patient infected with *Pneumocystis jirovecii* |

Supplementary Table 2: Indication and delay of prophylactic treatment after organ transplantation according to the different scientific societies
